# A Paradox in Bacterial Pathogenesis: Activation of the Local Macrophage Inflammasome Is Required for Virulence of *Streptococcus uberis*

**DOI:** 10.3390/pathogens9120997

**Published:** 2020-11-28

**Authors:** Nathan Archer, Sharon A. Egan, Tracey J. Coffey, Richard D. Emes, M. Filippa Addis, Philip N. Ward, Adam M. Blanchard, James A. Leigh

**Affiliations:** 1 School of Veterinary Medicine and Sciences, Sutton Bonington Campus, University of Nottingham, Loughborough LE12 5RD, UK; nathan.archer1@nottingham.ac.uk (N.A.); sharon.egan@nottingham.ac.uk (S.A.E.); tracey.coffey@nottingham.ac.uk (T.J.C.); richard.emes@nottingham.ac.uk (R.D.E.); adam.blanchard@nottingham.ac.uk (A.M.B.); 2 Advanced Data Analysis Centre, Sutton Bonington Campus, University of Nottingham, Loughborough LE12 5RD, UK; 3 Porto Conte Ricerche, 07041 Alghero, Italy; filippa.addis@unimi.it; 4 Dipartimento di Medicina Veterinaria, Università degli Studi di Milano, 20133 Milan, Italy; 5 Division of Structural Biology, Nuffield Department of Medicine, University of Oxford, Oxford OX3 7BN, UK; phil@strubi.ox.ac.uk

**Keywords:** *Streptococcus uberis*, mastitis, inflammasome, NLRP3, macrophage, pathogenesis

## Abstract

*Streptococcus uberis* is a common cause of intramammary infection and mastitis in dairy cattle. Unlike other mammary pathogens, *S. uberis* evades detection by mammary epithelial cells, and the host–pathogen interactions during early colonisation are poorly understood. Intramammary challenge of dairy cows with *S. uberis* (strain 0140J) or isogenic mutants lacking the surface-anchored serine protease, SUB1154, demonstrated that virulence was dependent on the presence and correct location of this protein. Unlike the wild-type strain, the mutant lacking SUB1154 failed to elicit IL-1β from ex vivo CD14+ cells obtained from milk (bovine mammary macrophages, BMM), but this response was reinstated by complementation with recombinant SUB1154; the protein in isolation elicited no response. Production of IL-1β was ablated in the presence of various inhibitors, indicating dependency on internalisation and activation of NLRP3 and caspase-1, consistent with inflammasome activation. Similar transcriptomic changes were detected in ex vivo BMM in response to the wild-type or the SUB1154 deletion mutant, consistent with *S. uberis* priming BMM, enabling the SUB1154 protein to activate inflammasome maturation in a transcriptionally independent manner. These data can be reconciled in a novel model of pathogenesis in which, paradoxically, early colonisation is dependent on the innate response to the initial infection.

## 1. Introduction

*Streptococcus uberis* is a widespread cause of intramammary infection in dairy cattle and a frequent cause of bovine mastitis worldwide. Treatment of bovine mastitis is a common reason for the administration of antibiotics within dairy production, representing approximately 30% of all antibiotic use attributed to cattle [1,2]. Mastitis reduces milk yields by 600–1200 kg per affected animal per year [3], creating inefficiencies resulting in the need for a greater number of farmed animals. Ruminants are also a common source of greenhouse gases, with estimates placing CO_2_e emissions between 1.03 and 1.3 kg CO_2_e L^−1^ (milk) [4] thus, inefficiency due to mastitis in the UK alone can be considered to contribute an additional 1 million tonnes of atmospheric CO_2_e/year. Consequently, infections with *S. uberis* pose an array of challenges to both animal welfare and sustainability in the dairy industry.

Following entry into the lactating bovine mammary gland, colonisation is dependent on replication in the extracellular environment, namely milk [5]. Virulent *S. uberis* strains typically reach more than 10^7^ cfu/mL milk, whereas avirulent or attenuated strains typically colonise less efficiently, suggesting a strong connection between colonisation and disease severity.

In most situations, initial control of bacterial infection is dependent on the innate immune response, which stimulates local inflammatory, bactericidal responses and subsequently recruits the adaptive immune response. The interleukin-1 (IL-1) family is broadly responsible for mediating these activities [6]: in a genome wide association study (GWAS) of *S. uberis* challenged dairy cattle the IL-1 gene cluster recently emerged as key immune modulators in bovine mastitis [7]. There are numerous examples of the role the initial host response plays in the control of streptococcal infections at mucosal surfaces [8,9].

Typically, epithelial cells contribute to the host immune response to a pathogen, enabling professional immune cells to be recruited to the site of infection [10,11]. In the same way, the mammary epithelial cells lining the bovine mammary gland respond to intramammary pathogens to initiate the host response and recruit neutrophils to the site of infection. However, several studies have noted that *S. uberis*, a commensal capable of asymptomatic carriage at many body sites of the dairy cow, failed to induce innate responses directly from bovine mammary epithelial cells [12,13]. Furthermore, transcriptional profiling of epithelium from animals infected with *S. uberis* was consistent with their stimulation by activated macrophages rather than direct stimulation by bacteria [14,15]. Further, a recent study demonstrated that although *S. uberis* failed to induce innate responses in vitro from primary mammary epithelium, it was able to induce innate responses from bovine blood-derived monocytes [12].

The study of *S. uberis* pathogenesis in the mammary gland offers unique insights for investigating host–pathogen interactions. In the mammary gland, host cellular defences can be considered to be in an inverted configuration compared to many tissues, with a sentinel leukocyte population located on the luminal side of the epithelial layer (within the secretion) rather than restricted to sub-epithelial layers. Resident milk leukocytes (predominantly macrophages and T lymphocytes in an uninfected gland) increase dramatically in response to infection, and the population shifts to one of predominantly neutrophils, which migrate to the site of infection in response to chemoattractant signals provided by resident leukocytes and epithelial tissues [16,17,18,19]. The neutrophil response to *S. uberis* is delayed compared to other mammary pathogens, which is consistent with the absence of a pathogen-induced innate signal directly from the epithelium, i.e., any signal from resident macrophages being diluted within the secretion. Colonisation by *S. uberis* has been shown to be unaffected by incoming neutrophils due to its ability to inhibit or resist their biocidal activities [20].

Pathogens are typically detected by the host via pattern recognition receptors (PRRs). These recognise pathogen-associated molecular patterns (PAMPs) such as lipopolysaccharides (LPS), dsRNA, lipoteichoic acid (LTA) [12], and muramyl dipeptide (MDP) [21], which originate from the pathogen [22,23,24]. This initiates a signalling cascade, guiding the formation of the inflammasome and generation of inactive “pro” forms of pro-inflammatory cytokines such as pro-IL-1β and pro-IL-18, thereby “priming” the inflammasome [25]. Following an initial “priming” event, host inflammatory responses are initiated or “activated”. This two-step model has been described widely [25]. First described in 2002 [26], several inflammasome complexes have now been characterised, including NLRP1, NLRP3, NLRC4, pyrin, and AIM2, with evidence emerging of further complexes such as NLRP6 and IFI16 [27]. These complexes detect specific pathogen- or damage-associated molecular patterns (PAMPs or DAMPs) such as extracellular ATP, particulate crystals [28] or the M protein of *S. pyogenes* [9,29] and recruit caspases to cleave pro-IL-1β and pro-IL-18 to generate mature, secreted pro-inflammatory cytokines. Detection of pro-inflammatory cytokines can also cause maturation of further inflammasomes in nearby cells, thereby amplifying the local inflammatory response. Pro-inflammatory cytokines elicit an influx of specialised cells, compounds, and other biocidal activities. Typically, these responses protect the host and are detrimental to the pathogen. However, the innate biocidal activities can also result in damage to the host [30], and thus, control over the inflammatory response to facilitate pathogen clearance whilst minimising host damage is required.

Inactivation of sortase (SrtA; a transpeptidase) responsible for covalent anchoring of proteins to the cell wall of *S. uberis* [31] impacted virulence. Subsequent analysis of individual SrtA substrate proteins indicated that colonisation and virulence were substantially reduced by inactivation of sub1154, which encodes a surface-anchored serine protease [32] with identity to a diverse family of high-molecular-weight, cell-surface-anchored (aka cell envelope) serine proteases of other pyogenic streptococci [33]. In the present study, we utilise this attenuated strain, along with isogenic counterparts, to characterise the role of SUB1154 during the early stages of pathogenesis using complementary in vivo and in vitro models.

## 2. Results

### 2.1. Streptococcus uberis Colonisation Requires SUB1154 Protein In Vivo

**Figure 1:**
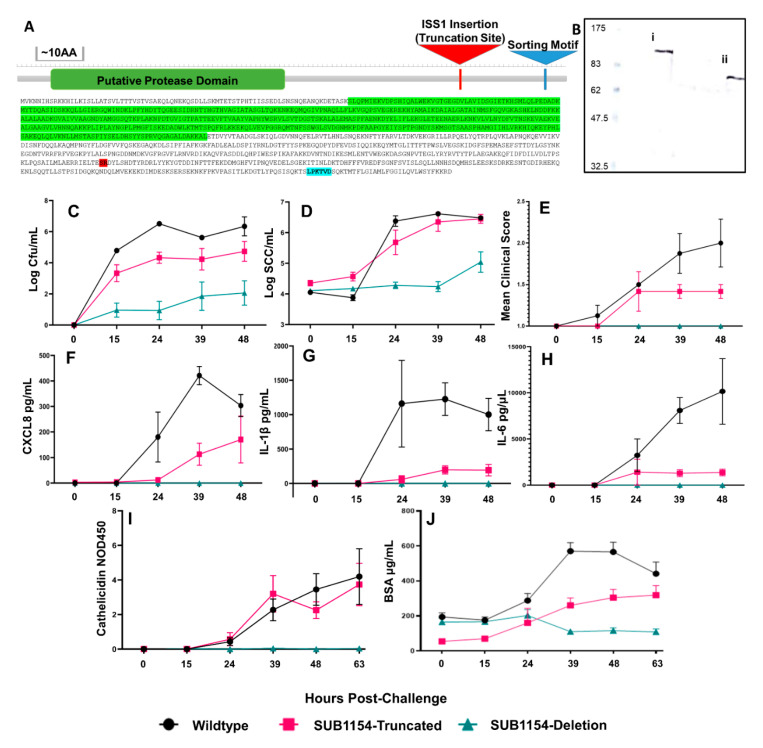
*Streptococcus uberis* pathogenesis requires SUB1154 protein in vivo. (**A**) Map of SUB1154 with putative active site (green), point of ISS1 insertion (red) present in the SUB1154-truncation, and the srtA anchoring motif (blue) annotated. Surface anchoring of SUB1154 and truncation of protein were confirmed by (**B**) immunoblotting, showing a detergent extract from SrtA mutant. *S. uberis* releases a full-length SUB1154 protein. (**i**) Extraction of protein from the media in which the SUB1154-truncation strain of *S. uberis* is grown releases an approximately 80 KDa protein (**ii**). (**C**) Colonisation (CFU/mL) measured from challenged quarters as colony-forming units isolated per mL milk (*p* = 0.0002) mirrored increases in somatic cell count (SCC/mL) (**D**) in response to SUB1154-truncated and wild-type strains (*p* < 0.0001). Only the wild-type SUB1154 expressing *S. uberis* strain (**E**) causes clinical manifestation (*p* = 0.0002). Enzyme-linked immunosorbent assay (ELISA) determined the concentration of CXCL8 (**F**) (*p* = 0.0005), IL-1β (**G**) (*p* = 0.0004), and IL-6 (**H**) (*p* = 0.0009) present in milk following challenge, showing that only the wild-type SUB1154 expressing *S. uberis* strain elicits a detectable and sustained inflammatory response. ELISA determined increases in the normalised OD450 levels of Cathelicidin (**I**) (*p* = 0.0008) and BSA (**J**) concentration (*p* ≤ 0.0001). Error bars are ± SEM. (**C**–**H**). Wild-type N = 4, SUB1154-truncated strain N = 6, SUB1154-deletion strain N = 6. (**I**,**J**): wild-type (**I** N = 10; **J** N = 8), SUB1154-truncated strain N = 6, and SUB1154 deletion strain N = 6. N = per quarter significances calculated with two-way ANOVA.

Mutants lacking the sortase-anchored serine protease, SUB1154, are attenuated in cattle [32]. We sought to characterise the role of this protein in bacterial pathogenesis. To this end, we challenged the bovine mammary gland with wild-type (WT) or previously generated isogenic *S. uberis* strains [32]. Briefly, a *sub1154* deletion (SUB1154-deletion) genotype was obtained by allelic exchange mutagenesis, whilst a truncated SUB1154 mutant (SUB1154-truncated) was isolated from a 0140J pGh:IS*S1* mutant bank as previously described [34]. The truncated SUB1154 protein lacks the sortase anchoring domain and is thus untethered from the bacterial cell surface (Figure 1A,B) but retains the putative serine protease domain.

In line with previous observations [32], SUB1154-deletion colonised the bovine mammary gland to a lower level (1000–10,000 fold) than the genetically intact WT strain (Figure 1C). SUB1154-truncated, expressing the non-anchored form of the SUB1154 protein, was better able to colonise compared to SUB1154-deletion but was still typically isolated in lower numbers (10–100 fold during the first 24 h) than the WT strain.

In response to infection, neutrophils migrate to the mammary gland, leading to a dramatic increase in the measured somatic cell count (SCC). As a result, SCC is a commonly used indicator of intramammary infection [35,36]. Neutrophils often comprise >90% of these cells present in the secretion of an infected mammary gland [37]. In agreement with this, increases in milk SCC were observed 24 h after challenge with bacteria expressing the SUB1154 protein, i.e., in response to both WT and SUB1154-truncated strains of *S. uberis* (Figure 1D). A less marked increase in SCC was apparent in response to the SUB1154-deletion strain.

Whilst bacterial colonisation and SCC were similar between WT and SUB1154-truncated strains, overt clinical signs (Table 1) were dependent on the presence of anchored SUB1154. Clinical signs were most severe in animals challenged with WT *S. uberis*, less severe in animals challenged with the mutant expressing the truncated-SUB1154 protein, and absent from animals challenged with the deletion mutant (Figure 1E). Initial colonisation and increased cellular influx appeared independent of anchoring of SUB1154, and severe clinical manifestation only occurred following challenge with the WT strain.

### 2.2. Host Cytokine Responses Reflect Overt Clinical Signs

Inflammatory and anti-inflammatory states are mediated by cytokines and chemokines to specify and recruit immune cells to the site of infection or repair. Canonically, the inflammatory response is detrimental to the pathogen. We thus sought to characterise the host response to *S. uberis* in our challenge study.

We performed ELISA on quarter milk samples collected prior to and at each milking after an intramammary challenge. Measurement of the chemokine CXCL8 (Figure 1F), pro-inflammatory cytokine IL-1β (Figure 1G), and pleiotropic IL-6 (Figure 1H) in vivo revealed no detectable cytokines until 24 h (h) post-challenge. The SUB1154-deletion strain yielded no detectable IL-1β response. The level of IL-1β remained substantially lower in response to SUB1154-truncated strain compared to WT. The IL-1β response was maximal to the WT strain by 24 h post-challenge and SUB1154-truncated strain at 39 h post-challenge. Similarly, whilst host CXCL8 chemokine levels were maximal in response to WT at 39 h post-challenge, CXCL8 increased for the full duration of the study (48 h) in response to the SUB1154-truncated mutant without reaching comparable maximal response. Challenge with the SUB1154-deletion did not yield detectable CXCL8. The IL-6 response (Figure 1H) to challenge with the WT did not show a maximal response but instead continued to rise through the post-challenge period. IL-6 responses to the SUB1154-truncated mutant was maximal at 24 h post-challenge and did not rise for the remainder of the study.

### 2.3. SUB1154 Elicits Host Antimicrobial Peptide Release and Reduces Epithelial Lining Integrity

Many intramammary infections are resolved by the bactericidal activities of macrophage and neutrophils [19,20,38,39,40,41]. These include production and release of acute-phase proteins (APP) and anti-microbial peptides (AMPs), which destroy, or inhibit growth of, bacteria [42]. Previous work observed the release of cathelicidin family members during *S. uberis* infection in cows [43]. Thus, seeking to measure the host antimicrobial responses to *S. uberis*, we measured cathelicidin levels in vivo from the milk of challenged animals (Figure 1I). The SUB1154-deletion strain did not elicit cathelicidin increases in the milk throughout the study, whilst SUB1154-truncated and WT strains both caused steadily increasing cathelicidin levels for the duration of the study.

Subsequently, we assayed bovine serum albumin (BSA) in milk (Figure 1J) as a marker for serum leakage, indicating changes to the integrity of the blood–milk barrier offered by the mammary epithelial lining [44]. We observed an increase in BSA concentration within milk when the gland was challenged with either strain expressing SUB1154. In response to WT, this increase peaked at the third milking post-challenge (39 h), and the response to the SUB1154-truncated strain remained lower than WT but increased for the duration of the study. Challenge with the deletion strain elicited no increases in BSA concentration.

For the pathogenicity and host parameters measured (Figure 1C–J), we observed that responses to the wild type were maximal (indicating greater colonisation, host response, and disease) and that these were reduced if SUB1154 was truncated, and thereby not anchored, and absent if the SUB1154 protein was missing. As mastitis is a disease with an inflammatory pathology (in response to bacterial infection), it is logical that the cascade of events matches the host response to the colonising bacterium. However, differences in colonisation (up to 10,000-fold) were clearly detected at the first milking post-challenge and prior to any detectable changes in any other parameter (Figure 1C–J). The WT and SUB1154-deletion strains have been shown to grow equally in raw bovine milk and Todd Hewitt broth [32], and these strains have been shown to share identical (except for the introduced deletion) genome sequences (data not shown). This led to the supposition that differences in early colonisation resulted from subtle or localised events, the consequences of which were not apparent or detectable following dilution in the entire milk volume.

### 2.4. SUB1154 Elicits IL-1β Release from a Novel Ex Vivo Challenge Model in an NLRP3-Dependent Manner

Although mammary epithelial cells are involved in the overall response to *S. uberis* [12,45,46], in vivo, during clinically apparent disease, their transcriptional programmes are more consistent with indirect stimulation via macrophage that have themselves been activated [14,15]. Hitherto, our data show that only SUB1154-expressing (WT and SUB1154-truncated) *S. uberis* trigger pro-inflammatory cytokine release in vivo. We therefore sought to investigate the likely first-responding cells, mammary macrophages, in vitro.

**Figure 2:**
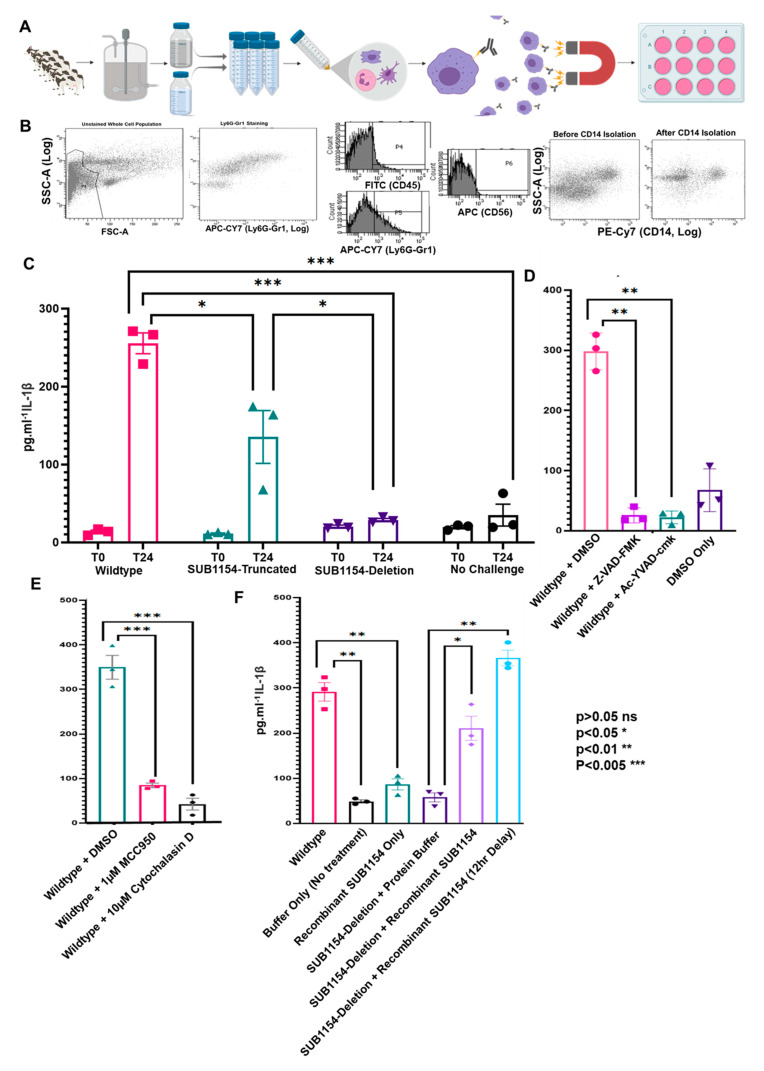
SUB1154 mediates the caspase-dependent maturation of IL-1β. (**A**) Schematic of CD14^+^ cell isolation from raw bovine milk, enabling assay of proteomic and transcriptomic responses in vitro. The rich population of cells isolated from milk was then visualised by flow cytometry (**B**) scatter plots including a population of neutrophils as stained by Ly6G-Gr1 (representative of four replicates). This population complexity was reduced following isolation of CD14^+^ cells by Flowcomp beads. Following enrichment of CD14^+^ cells, ELISA determined the IL-1β, responses of the CD14^+^ enriched cells to *S. uberis* WT, Sub1154-Deletion, and Sub1154-truncated strains (**C**). Expanding this model by caspase inhibition (**D**) by either Z-VAD-FMK or Ac-YVAD-cmk on IL-1β during CD14^+^ cell challenge prevented normal IL-1β response to wild-type *S. uberis* during the 24 h (*p* = 0.0013 and 0.0017 respectively). Inhibition of NLRP3 oligomerisation or endocytosis by MCC950 and Cytochalasin D, respectively, drastically reduced IL-1β secretion in response to wild-type *S. uberis* (**E**). Recombinant SUB1154 protein was unable to trigger the release of IL-1β alone from ex vivo milk CD14^+^ cells. Concurrent incubation with the deletion strain recapitulated a wild-type-like response. (**F**). Error bars are ± SEM, and *p*-value was calculated with students *t*-test and denoted by *, **, or *** in the figures. Two-way ANOVA on (**C)** *p* = 0.0001, (**E**) *p* ≤ 0.0001, one way ANOVA on (**D)** *p* ≤ 0.0001, **F** *p* ≤ 0.0001, (**C**–**F**); N = 3 for each treatment.

Previous models for macrophage challenge have been generated from bovine-blood-derived monocytes (boMdM) or murine cell line RAW 264.7. Whilst boMdM avoids the issues of being immortalised in vitro when used and is bovine in origin, extracting cells from raw bovine milk (Figure 2A) allowed us to generate an ex vivo model from the same population of cells normally present in the bovine mammary gland.

A rich, heterogeneous population of leukocytes was extracted from milk (Figure 2B), and the enrichment of bovine CD14^+^ cells using magnetic beads enabled the generation of a convenient model for in vitro challenge of ex vivo macrophage-enriched cells (Figure 2A). Initially, CD14^+^ isolation results in two populations as neutrophils express CD14 to a much lower extent; however, only macrophages are adherent and thus the act of seeding isolated CD14+ cells on plastic further enriches macrophages.

Following challenge in vitro with heat-killed WT SUB1154-truncated and SUB1154-deletion strains, we performed ELISA for IL-1β. Differences in host responses observed in WT SUB1154-truncated and SUB1154-deletion strains in vivo were recapitulated in vitro with isolated ex vivo CD14^+^ cells (Figure 2C), highlighting that the SUB1154-dependent IL-1β effects seen in vivo are not simply an effect of increased numbers of colonising bacteria. Next, we performed the challenge under caspase inhibition, in the presence of the pan-caspase inhibitor Z-VAD-FMK and the selective caspase 1 inhibitor Ac-YVAD-cmk, and both treatments ablated IL-1β response to WT *S. uberis* (Figure 2D). Subsequently, we performed the challenge in the presence of MCC950, which targets the NLRP3 NATCH domain and prevents its oligomerisation and thus acts, specifically, to prevent NLRP3 inflammasome activation [47]. This resulted in a >4-fold reduction in IL-1β following challenge (Figure 2E). Finally, we performed the challenge in the presence of Cytochalasin D, which prevents many forms of endocytosis, including phagocytosis and macropinocytosis. This resulted in a >8 fold reduction in IL-1β in response to challenge (Figure 2E).

### 2.5. SUB1154 Alone Does Not Elicit IL-1β from Naïve Macrophage

We investigated whether the SUB1154 protein alone could initiate secretion of the pro-inflammatory cytokine IL-1β from the macrophage present in milk. To answer this, we incubated ex vivo bovine CD14^+^ enriched cells with recombinant SUB1154 protein (Figure 2F). Recombinant SUB1154 protein alone was unable to stimulate release of IL-1β. By way of a complementation study, we demonstrated that simultaneous co-stimulation with SUB1154 and the SUB1154 deletion strain partly restored the IL-1β response (Figure 2F) to levels similar to those of responses to WT. Following the two-signal model of inflammasome activation [48], we postulated that the recombinant SUB1154 protein would activate the inflammasome in CD14^+^ cells otherwise primed by an initial *S. uberis* signal; in this context, *S. uberis* lacking the SUB1154 protein. Therefore, in the same series of experiments, we added recombinant SUB1154 protein 12 h after stimulation with, and removal of, the SUB1154-deletion strain. This resulted in a recapitulation of the responses to levels very similar to WT.

### 2.6. The Host Transcriptome Is Reprogrammed in Response to S. uberis Independently of SUB1154

Our ex vivo model allows for the assay of RNA and extracellular protein from the same sample. Thus, exploiting our unique ex vivo macrophage-enriched model, we sought to understand the transcriptional profiles of host cells 24 h after *S. uberis* challenge and then to disentangle the effect of the wild-type SUB1154 protein.

We interrogated transcriptomes of ex vivo CD14^+^ cells responding to WT or SUB1154-deletion strains of *S. uberis* by next-generation sequencing. Following challenge, there is likely contamination of mammalian mRNA with *Streptococcus* RNA species, where the abundant ribosomal RNA is polyadenylated [49]. To account for these, we used “Quant-seq 3′ FWD” for Illumina, capturing only the portions of transcripts proximal to the poly(A) tail, reducing the required depth and cost of sequencing and then correcting for PCR bias using unique molecular indexes (UMIs) added to each strand during second-strand synthesis [50,51].

**Figure 3:**
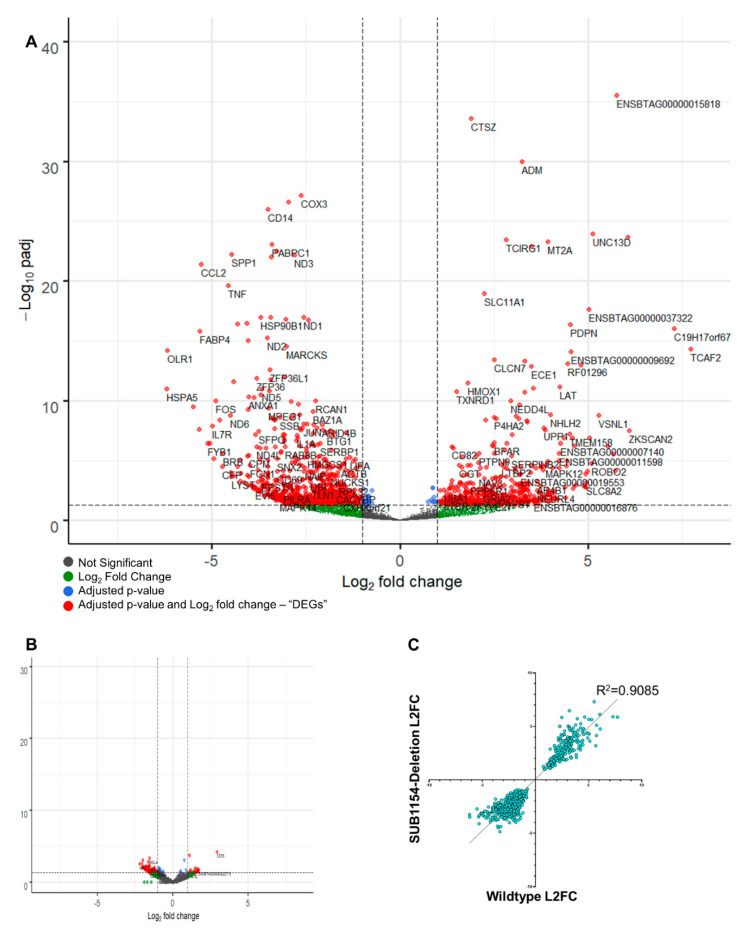
The transcriptional programme changes independent of SUB1154. (**A**) Volcano plot showing the transcriptional changes during responses to wild-type *S. uberis.* X-axis is L2FC, *Y*-axis is adjusted *p*-value. Significantly different (*p* ≤ 0.05, l2 fc ≥ 1.0 or ≤−1.0) genes are shown in red. Green signifies genes with detected non-significant differences (*p* ≥ 0.05). Grey signifies no change beyond threshold values. Plotting the differential gene expression analysis of 24 h time points of CD14^+^-enriched cells responding to WT or SUB1154-deletion on the same scale exhibits the minimal differences between transcriptome programmes (**B**). Directly comparing the log2 fold-change values between T0 and T24 for responses to wild-type or SUB1154-deletion for every gene with an adjusted *p*-value < 0.05 in both datasets shows a strong correlation (**C**).

Differential expression analysis (Figure 3A) detected 1052 differentially expressed genes (DEGs) with a Benjamini–Hochberg-adjusted *p*-value ≤ 0.05 and log_2_ fold change of ≥1.5 or ≤−1.5 in response to WT *S. uberis* 24 h following challenge; 902 DEGs were detected in response to the SUB1154 deletion strain of *S. uberis*. Broadly, similar changes to transcriptomes were detected in response to both wild-type and deletion (Figure 3B,C)

**Figure 4:**
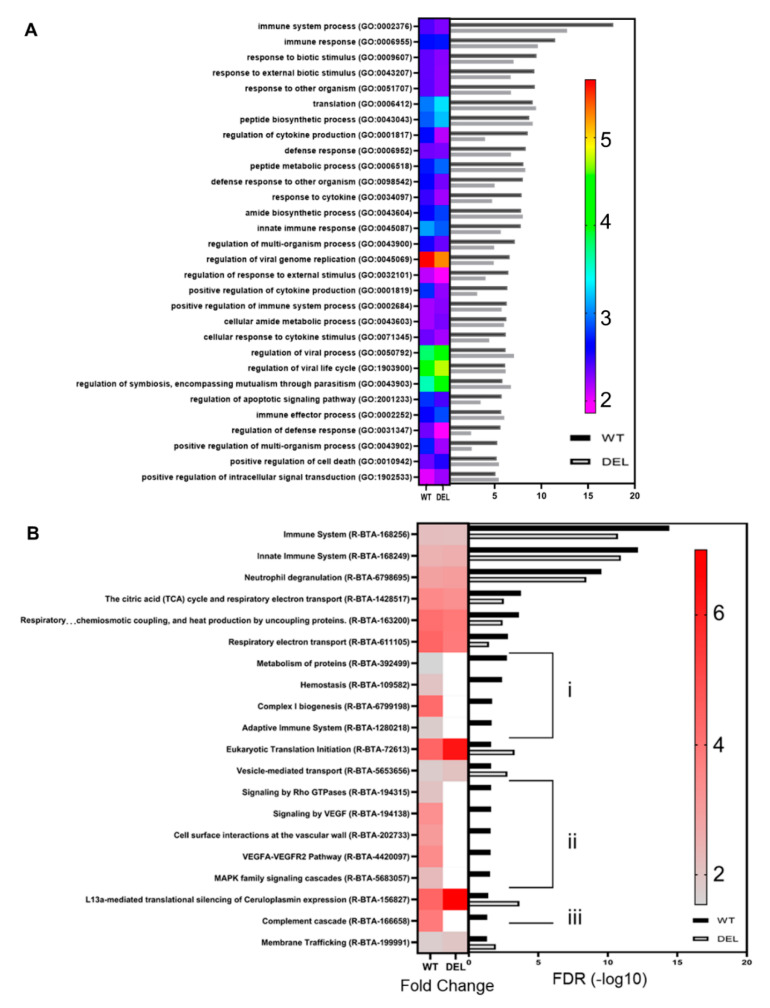
Functional categorisation of enriched genes and pathways. (**A**) GO term heatmap shows fold enrichment as a heatmap of colour with FDR plotted as a bar chart for each term. Full list of terms available in supplemental data. Similarly, reactome enrichment (**B**) showed enrichment for genes associated with immune system processes and translation, with enrichment largely independent of sub1154 genotype. Several terms were enriched only in the wild-type DEGs (**i**), (**ii**), (**iii**). Full list of terms available in supplemental data (Tables S1 and S2).

GO-enrichment on DEGs highlighted numerous terms (Figure 4A, Tables S1 and S2) associated with the changes in transcriptional programme, including immune response (GO:0002376), response to other organisms (GO:0051707), regulation of cytokine production (GO:0001817), and defence response (GO:0006952). These terms had similar enrichment in the response to SUB1154-deletion (Figure 4A).

Taking only enriched DEGs (≥+1.5 l2 fc and padj ≤ 0.05), we detected a >3-fold enrichment of inflammatory response GO:0006954 (FDR 0.0373). Genes associated with GO:0006954 changed similarly between challenge groups; however, IDO1, important in the modulation of Th17 cell recruitment [48], dendritic cell activity, and tryptophan degradation [52], was notably increased in response to WT but decreased in response to deletion (Figure 3A and Figure S2), suggesting that it is a SUB1154-dependent DEG. Reactome enrichment on DEGs revealed enrichment of immune system (R-BTA-168256), Neutrophil Degranulation (R-BTA-6798695), and translation-associated terms, amongst others (Figure 4B). Interestingly, several of the most significant terms enriched in response to WT *S. uberis* were not significant in response to deletion, including adaptive immune system (R-BTA-1280218) (Figure 4Bi), MAPK family signalling cascades (R-BTA-5683057) (Figure 4Bii), and complement cascade (R-BTA-166658) (Figure 4Biii), suggesting that it is the downstream processing following immune recognition, which is dependent upon the presence of SUB1154.

### 2.7. Downstream Inflammasome Signalling Characterises the SUB1154-Dependent Host Response

**Figure 5:**
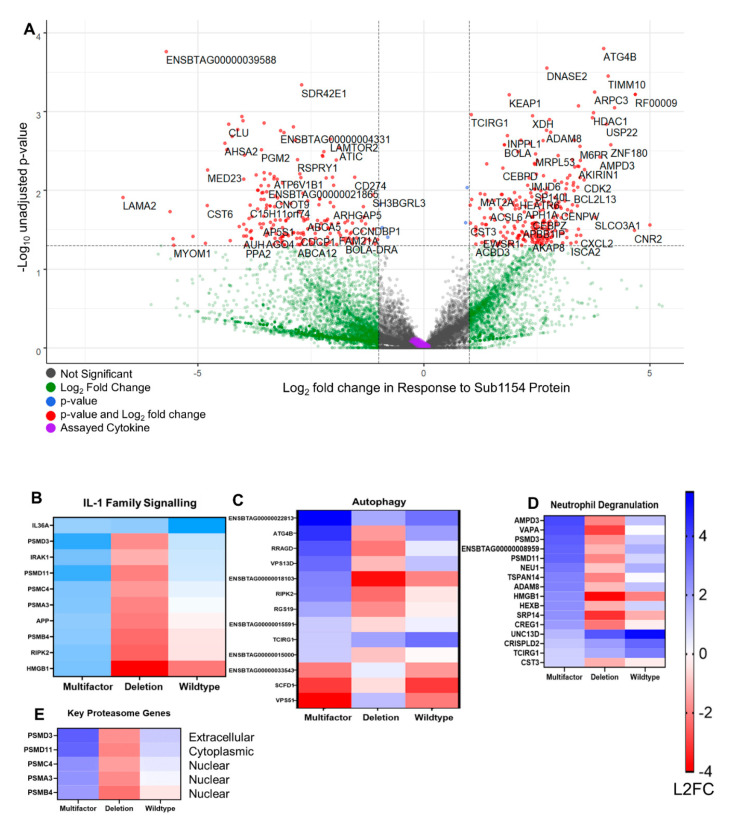
Transcriptional programming dependent on SUB1154 protein. (**A**) Multifactor analysis revealed the transcriptional programme changes caused specifically by the presence of SUB1154 protein (i.e., ATG4B is overexpressed in the presence of *S. uberis* expressing SUB1154 protein). Genes in red were then used for subsequent reactome or GO term enrichment. Cytokines assayed in Figure 1; Figure 2 are shaded in purple. Heatmaps were drawn to disentangle DEGs from those that are specifically regulated in response to SUB1154 protein. GO terms IL-1 Family Signalling (**B**), Autophagy (**C**), and Neutrophil degranulation (**D**) were chosen as they have significant FDR values in Table S3. Key proteasome genes (**E**) were responsible for several GO terms (See also Figure S3). Colour intensity denotes log_2_ fold-change, and all genes shown were significant (unadjusted *p*-value ≤ 0.05) in the multifactor analysis.

We queried changes in the transcriptional programme caused by the presence of sortase-anchored SUB1154 protein on *S. uberis* by carrying out both a “simple” A_B comparison between 24 h time points of both challenges and a multifactor differential expression analysis, building a model that takes into account the expression levels in ex vivo cells at T0. Broadly, we found minimal transcriptome changes to be dependent on SUB1154 (Figure 4B,C). Notably, IL-1β and CXCL8 were not SUB1154-dependent DEGs (Figure 5A and Table S3), suggesting that the SUB1154-dependent effects on cytokine secretion are post-transcriptional. Indeed, broadly, the transcriptional programmes were very similar at 24 h, with no SUB1154-dependent genes gaining a Benjamini–Hochberg-adjusted *p*-value ≤ 0.05, thus further implicating SUB1154 in the final caspase-dependent maturation of the inflammasome “activation”, which is largely independent of transcription for the secretion of IL-1β [53].

Reactome enrichment on SUB1154-dependent DEGs with unadjusted *p*-values ≤ 0.05 corroborates recent identification of the IL-1 gene cluster as a mastitis-modulating region [7]. This also revealed >8-fold enrichment of “Interleukin-1 family signalling”, alongside other terms such as “Class I MHC mediated antigen processing and presentation”, “p75NTR signals via NF-κB”, and “Neutrophil degranulation”, amongst others (Figure 5B–E and Table S3). Many upregulated terms were the result of an enrichment of proteasomal genes PSMD3, PSMA3, PSMB4, PSMC4, and PSMD11 (Figure 5E) and the proteasome complex GO term following network analysis (padj 8.31 × 10^−49^) (Figure S3). Several genes associated with the KEGG term “cytokine-cytokine receptor interaction” were SUB1154-dependent alongside several associated with cell adhesion molecules (Figure S4). Genes with lower expression in response to WT than deletion did not reach FDR-corrected significance for any complete GO-term.

## 3. Discussion

Combining in vivo and in vitro study, we investigated the early pathogenesis of *S. uberis* as it colonises the bovine mammary gland. *In vivo*, we confirmed earlier observations [32] that SUB1154 was required for virulence and optimal colonisation of the mammary gland. Furthermore, we demonstrated that cell surface anchoring of the SUB1154 protein was required for full virulence. This latter observation is consistent with a previous investigation of a *srtA* mutant of this strain, in which, similarly to the strain expressing the truncated SUB1154 in this study, it was able to colonise and induce a cellular infiltration in the absence of clinical signs of disease [32]. These data reflect the necessity of SUB1154 and its association with the bacterial cell as a virulence factor, which is further underpinned by the ubiquitous presence of full-length sub1154 sequences containing the SrtA anchor motif in mammary isolates of *S. uberis* [54,55]. Given the improved in vivo growth, and similar SCC response observed in response to SUB1154-truncated, it seems likely that the truncated gene product retains activity, although this cannot be verified in the absence of defined substrate protein.

We extended these observations by measuring the host response to infection. Both our in vivo and ex vivo observations showed that SUB1154 was required to initiate an innate inflammatory response by the host, establishing that, whilst colonisation corresponded with the presence of SUB1154, the same protein also acted as a trigger for the host response to infection. Colonisation of the bovine mammary gland by *S. uberis* continued despite biocidal activities of the host, confirming many previous observations that virulent strains of this pathogen are not deterred by the host inflammatory defences [20,38].

Utilising an ex vivo model enabled the assay of cells extracted from the target organ in vitro, we corroborated the findings in vivo that IL-1β release was dependent upon the SUB1154 protein and not simply the result of a canonical inflammatory response to different numbers of colonising bacteria. Further, these data corroborate previous reports that *S. uberis* induces innate immune responses from bovine peripheral blood monocytes and murine macrophage cell line Raw 264.7 [13]. Performing challenges in the presence of small molecule inhibitors highlighted that the maturation of IL-1β induced by wild-type *S. uberis* is likely mediated by Caspase 1, as Ac-YVAD-cmk is a highly selective inhibitor of caspase 1, with some activity against caspase 4 [54,55], (although its activity against the bovine-specific caspase 13—an orthologue of caspase 4—is unknown). We also showed that SUB1154-dependent activation is dependent upon the oligomerisation of NLRP3, which recruits pro-caspase 1 [9,23,56]. These studies also demonstrated the utility of our ex vivo model in screening small molecule inhibitors for their ability to attenuate responses in milk leukocytes, which can be prepared non-invasively.

Although we detected several important transcriptome changes in the responding CD14^+^ cells, we detected minimal SUB1154-dependent DEGs. Those which were SUB1154-dependent were typically associated with the downstream adaptive immune system and/or inflammatory processes, including the IL1 gene cluster, which has previously been associated with severity of mastitis through GWAS of several hundred animals experimentally challenged with another strain of *S. uberis* [7].

Phagocytic uptake of *S. uberis* by macrophage has been shown in vitro [57] and in vivo [58]. This would enable *S. uberis* to gain access to the intracellular environment, although not necessarily the cytoplasmic compartment. In our study, we demonstrated that production of IL-1β was ablated by cytochalasin D, which prevents endocytosis, including phagocytosis and macropinocytosis. Clearly, our studies indicate a direct or indirect interaction between host NLRP-3 and/or other inflammasome components and SUB1154 (or its products), and further detailed studies building on our initial observations would be required to fully elucidate this molecular mechanism.

In vitro, we were able to recapitulate the IL-1β response of CD14^+^ cells to the strain lacking SUB1154 by supplementing with recombinant SUB1154, showing that the SUB1154 protein can act somewhat independently of the bacteria on otherwise primed CD14^+^. It remains to be seen if the addition of this exogenous SUB1154 protein also requires internalization; however, this would be postulated if SUB1154 directly targets NLRP3 or other inflammasome related cytoplasmic proteins.

A model in which a pathogen stimulates an inflammatory response that enhances colonisation and disease pathology is not unique but represents a break from the canonical [56], whereby pathogens evade or subvert early host immune signalling [59]. For example, cytokine exposure may increase the growth of uropathogenic *E. coli* [56], whilst inhibiting the host inflammatory response can prevent mortality associated with *Bacillus cereus* [60]. In contrast, *Staphylococcus aureus* directs host macrophage towards an anti-inflammatory polarisation [61]. Since in response to *S. uberis* we measured in vivo increases in levels of CXCL8, a pro-inflammatory chemokine involved in the recruitment and degranulation of neutrophils [18,62], and detected evidence of a pro-inflammatory response in our ex vivo model, our data do not support *S. uberis* exhibiting an anti-inflammatory constraint on the host.

Conversely, streptococci appear to actively evoke immune responses: the inflammasome activating M1 protein [9] enables the sequestration of cathelicidin [63] and is responsible for lung damage in humans, which was recently shown to be mediated by neutrophil degranulation [30]. The GAS protease SpeB directly cleaves IL-1β, initiating the host biocidal activities to disrupt the host microbiota during nasopharynx infection [64]. Further, when exposed to LL-37, a human cathelicidin, *S. pyogenes* drives an increase in the host inflammatory response [65]. In the case of *S. uberis*, we and others [43] have shown that challenge in ruminants results in a pro-inflammatory state, leading to cathelicidin release. Notably, many virulent streptococci are resistant to the microbiocidal effect of cathelicidins [63,66]. In contrast to other mastitis-causing agents, *S. uberis* has been shown to resist host defences in vitro and in vivo [20,67,68], including resisting the bactericidal action of neutrophils [38]. These microbiocidal activities also typically damage the host [30]; however, an inflammasome-independent activity of NLRP3 was shown to preserve the alveolar epithelial cell integrity during *Streptococcus pneumoniae* infection in a mouse model [8]. This defence may be unavailable in the bovine mammary gland as *S. uberis* appears not to directly trigger epithelial cell responses [12], which are instead initiated by activated macrophages [14,15]. Indeed, we measured a loss of epithelial integrity, as the increased presence of BSA in milk indicated a breakdown of the blood–milk barrier and consequent serum leakage into the mammary gland. Our data fit an emerging model of streptococci pathogenesis, during which disease progression is promoted (rather than hampered) by innate inflammatory changes and demonstrate a new mediator of this process, the *S. uberis* cell envelope serine protease, SUB1154. This protein appears to be able to act on host immune cells that have already been primed through an alternative route. This may suggest that prior, distinct inflammasome-priming events could encourage initial colonisation of the mammary glands with *S. uberis*, and we speculate whether such an event may also underpin opportunistic infection by other streptococci in other situations.

These events can be reconciled if one considers that the damage caused by the host’s own biocidal activities enhance the availability of nutrients for the colonising *S. uberis.* We have previously measured an increased microbial growth (rate and yield) in serum-supplemented milk (unpublished data [69]), showing this to be a plausible explanation. This host damage, along with increasing numbers of bacterial cells, would provide more PAMPs and DAMPs, resulting in a continually increasing series of diverse inflammatory stimuli [25]. Despite the removal of around 95% of total gland content (secretion) at milking, this spiralling cycle of inflammasome activation and further amplification of inflammatory signals by increasing bacterial numbers, pyroptotic macrophage, and degranulating neutrophils [70] leads to the inevitable consequence of hyper-inflammation that constitutes mastitis.

In summary, we show that SUB1154, a cell envelope serine protease, induces a potentially biocidal environment by stimulating the NLRP3 inflammasome, which in the absence of pathogen elimination leads to an increased inflammatory response. At this point, the increasing bacterial number and consequent inflammatory stimulus mean that without intervention, the characteristic inflammatory pathology of mastitis is inevitable. This proposed model of early pathogenesis thus intimately connects *S. uberis* virulence and mammary gland colonisation with the initial host response. Our model expands on an emerging phenomenon in streptococcal infections, which sees the earliest host response playing a critical role in enabling or inhibiting early colonisation. Our data also inform the growing interest in the multifunctional serine proteases commonly located on the surface of pyogenic streptococci. Here we present a new functional role: eliciting an inflammatory response.

## 4. Materials and Methods

### 4.1. Isolation of CD14^+^ Leukocytes from Bovine Milk

Milk leukocytes were initially isolated in a similar fashion as described previously [71]. Briefly, milk was collected from a bulk tank, and somatic cell count was determined using a DeLaval portable cell counter—a preparation would not continue if this exceeded 100 SCC/µL (representing 100,000 SCC/mL), and a typical starting concentration ranged from 30–50 SCC/µL. Milk was collected into sterile glassware containing an equal volume of PAE buffer comprising 10% acid-citrate dextrose, 20 mM EDTA, and 0.02 volumes of antibiotic–antimycotic (100×) in PBS. Centrifugation, with no braking, at 700× *g* for 40 min at 15 °C was performed before the top layer of fat was removed and the supernatant aspirated. The leukocyte pellet was resuspended, washed once with PAE by centrifugation at 500× *g* at 5 °C, and resuspended in isolation buffer consisting of PBS supplemented with 0.1% BSA and 2 mM EDTA. The cell numbers were estimated using the DeLaval portable cell counter in line with the manufacturer’s instructions and adjusted to 5 × 10^7^ per mL.

CD14^+^ cells comprise approximately 15–30% of the total cell population found in milk and were isolated using Dynabeads flowcomp human CD14 kit (Invitrogen) to isolate bead-free CD14^+^ monocytes. The manufacturer’s instructions for isolation from buffy coat were followed after ex vivo milk leukocytes were resuspended in isolation buffer consisting of PBS supplemented with 2 mM EDTA and 0.1% BSA. Ex vivo cells were incubated with 5 µL of the included human CD14 antibody per 1 mL of cell suspension for 10 min on ice before proceeding with the remainder of the manufacturer’s instructions. 

Typically, 2 L of milk would yield approximately 1–2 × 10^7^ CD14^+^ cells, enabling all 12 wells of a tissue culture dish to be seeded at 0.5 × 10^6^ cells/mL (1 × 10^6^ per well) and recovered overnight at 37 °C and 5% CO_2_ in growth media consisting of IMDM media supplemented with L-glutamine, 20% FBS, and Antibiotic-Antimycotic (Gibco). Following seeding onto tissue culture dishes, each well was considered a biological replicate, with all treatments of a study performed from a single batch of milk collected on the same day. After 16 h, wells were observed for signs of bacterial or fungal growth and discarded if apparent. Adherent CD14^+^ cells were washed twice with pre-warmed PBS, also removing any remaining contaminating leukocytes, and 1 mL of fresh growth media was added to each well. CD14^+^ cells were then challenged after 1 h in fresh media.

### 4.2. Bacterial Strains and Culturing Conditions

*S. uberis* strain 0140J (strain ATCC BAA-854/0140J), originally isolated from a clinical case of bovine mastitis in the UK, was used throughout this study. Strains were routinely grown in Todd Hewitt (THB) broth (Oxoid Ltd., Cambridge, UK) at 37 °C. The SUB1154-deletion mutant was generated as previously described, and the SUB1154-truncated mutant was isolated from a bank of *S. uberis* 0140J pGh9::IS*S1* mutants following PCR screening using a primer specific for the *sub1154* locus (SUB1154 fwd 5′-GAAATGATGATGAGAAATTGAGA-3′) in conjunction with those specific to ISS1 (ISS1 fwd 5′-GCTCTTCGGATTTTCGGTATC-3′; ISS1 rev 5′-CATTTTCCACGAATAGAAGGACTGTC-3′) as described previously [32].

### 4.3. Detergent Extraction and Immunoblotting of SUB1154

*S. uberis* strain 0140J, mutant for SrtA, and SUB1154-deletion were pelleted by centrifugation at 8000× *g* for 6 min before being washed thrice in PBS. Pellets were resuspended 200 µL of 0.1% (*v*/*v*) Nonidet P-40 (NP40) (ThermoFisher ScientificLoughborough, UK), and detergent extract was harvested following removal of the bacterial cells by centrifugation at 16,000× *g* for 10 min at 4 °C.

Immunoblotting was performed as before [32]. Briefly, detergent extracts were separated on 10% SDS-PAGE gels before transfer onto a nitrocellulose membrane (Amersham) at 170 mA for 1 h in transfer buffer (25 mM Tris-base, 192 mM glycine, and 20% *v*/*v* methanol, pH 8.1). Blocking with 1% skim milk powder in PBS was then performed at 4 °C overnight. The membrane was washed thrice with PBS containing 0.1% Tween-20 (PBST) prior to incubation with rabbit antisera raised to SUB1154 (Davids Biotechnologie, Regensburg, Germany) in blocking solution at 1:12,000 dilution for one hour. Membranes were washed three times for 5 min with PBST and then incubated with goat anti-rabbit igG conjugated to HRP (Southern Biotech) at a 1:1000 dilution. Following a further three washes in PBST, HRP conjugate was detected using a solution of 4-chloronaphthol (0.5 mg mL^−1^) in PBS containing 16.7% methanol and 0.00015% (*v*/*v*) H_2_O_2_ and a one-hour incubation in the dark before membranes were washed in PBS and allowed to dry before imaging.

### 4.4. Animal Models of Bovine Mastitis

To determine the host responses to infection with the *S. uberis* strains, dairy cows 2–10 weeks into their first lactation were selected for experimental challenge. Criteria for selection included the absence of signs of mastitis, absence of bacteria in milk samples taken 24–48 h prior to challenge, no history of mastitis during the current lactation, and no evidence of intramammary infection in milk samples taken at 7 to 14 days after parturition and 24 h prior to challenge. Animals would have been retrospectively excluded from the study if high levels of immune-modulating cytokines (IL-1β, CXCL-8, or IL-6) were detected (none were), or not used in the study if somatic cell counts of greater than 100,000 cells/mL were detected in pre-challenge milk samples, as these may indicate a subclinical infection.

### 4.5. Ethics Statement

The challenge study was conducted at the Institute for Animal Health (now Pirbright Institute), Compton Laboratory, in accordance with the UK Animal Scientific Procedures Act under the project licence PPL:30/2645.

### 4.6. Intramammary Challenge with S. uberis Strains

Eight dairy cows in total (Holstein Friesian) were challenged in two contralateral mammary quarters each after PM milking by infusion of 1 mL of pyrogen-free saline (Sigma, UK) containing 1–2 × 10^3^ CFU of *S. uberis* strain WT, SUB1154-truncated, or SUB1154-deletion. Challenge dose was grown overnight, washed thrice and diluted accordingly. Two cows (four quarters) received WT, four cows (eight quarters) were challenged with SUB1154-Deletion, and four cows (eight quarters) were challenged with SUB1154-Truncated. Each quarter was treated as a biological replicate in line with established models [16,32].

Milk samples were obtained twice daily at the routine (a.m. and p.m.) milkings, thus conducted at 9 h (between a.m. and pm) and 15 h (between p.m. and a.m.) intervals as shown in Figure 1. To estimate viable bacteria concentrations, the milk was transported at 4 °C prior to plating of dilutions of each milk sample onto blood agar containing 1% (*w*/*v*) aesculin (ABA), and the number of somatic cells present in milk was measured using a DeLaval portable cell counter in line with the manufacturer’s instructions.

Strain-specific PCR amplification was performed on the recovered bacterial isolates to confirm the altered gene locus.

### 4.7. Clinical Scores

Clinical assessment of animals was undertaken at each milking. In the context of the current study, assessment of milk quality and udder inflammation was undertaken according to previously described criteria [32] (Table 1) and clinical endpoints. Animals were scored blind by a herdsperson lacking knowledge of which cows or quarters were challenged and with what strain.

Following the 4th milking post-challenge (48 h), all challenged quarters received intramammary antibiotic therapy and were discharged from the study once infection was cleared.

### 4.8. ELISA Analysis of Milk Samples

Quarter milk samples (whole milk) were analysed by ELISA against standards of known concentration in triplicate. Commercially sourced kits to detect the presence of bovine BSA (Bethyl laboratories) and bovine IL-1β and IL-6 (Thermo Scientific, UK) were used as described in the manufacturers’ instructions.

The concentration of CXCL8 was determined by ELISA, using human IL-8 specific antibodies (R&D Systems) and a modified luminescence-based ELISA with all reagents tested at a volume of 100 µL/well. Briefly, black 96-well immunoplates (Thermo Scientific, UK) were coated (overnight at 4 °C) with monoclonal capture antibody MAb208 (2 µg/mL) in 0.05 M carbonate/bicarbonate coating buffer (pH 9.6; Sigma). Between incubations, plates were routinely washed 5 times with PBS containing Tween-20 (1% *v*/*v*) PBST. Unsaturated binding sites were blocked for at least 1 h with a solution of 1 mg/mL sodium casein in PBS. Samples were diluted where appropriate with PBS, and bovine recombinant CXCL8 (Kingfisher Biotech Inc., St Paul, MN, USA) was used to generate an appropriate standard curve. Detection antibody BAF208 was used at a concentration of 0.1 µg/mL, Streptavidin-HRP antibody (GE-Healthcare Lifesciences) at 1:500 dilution, and luminescence measured using the Super Signal ELISA femto maximum-sensitivity substrate (Pierce).

Differences between groups along timepoints were assessed by repeated measures two-way analysis of variance (ANOVA).

Cathelicidin levels were assessed at the Porto Conte Ricerche laboratories with an in-house sandwich ELISA based on two pan-cathelicidin monoclonal antibodies, as detailed previously [72]. Normalized OD450 value (NOD450) was obtained by subtracting from the optical density at 450 nm (OD450) of all experimental samples the average OD450 (+3 SD) of 6 healthy control quarter milk samples with <50,000 cells/mL.

### 4.9. Flow Cytometry

Flow cytometry was performed one day after initial extraction and seeding, though the seeding onto plastic and further wash steps performed prior to challenge would further enrich the adherent macrophage. Whole-cell and isolated cell types were stained with FITC-conjugated CD45 to stain all leukocytes in the population, APC-conjugated CD56 (Monoclonal, MEM-188; Invitrogen, Carlsbad, CA, USA), as an NK cell marker, and APC-Cy7-conjugated Ly-6G/Gr-1 (Monoclonal, RB6-8C5; Invitrogen, Carlsbad, CA, USA) as a neutrophil marker. Human CD14 PE-Cy7 (Monoclonal, M5E2; Fisher Scientific, UK) was used following overnight recovery of the cells. Flow cytometry was performed on a BD FACS Canto II using the FACS Diva Software to image populations as scatter plots. A scheme of doublet discrimination and gating is available in Supplemental Data (Figure S5).

### 4.10. Cytokine Response of Ex Vivo CD14^+^ Cell Challenged with S. uberis and Recombinant SUB1154 Protein

0140J WT, SUB1154-truncated, and SUB1154 deletion strains of *S. uberis* were cultured overnight at 37 °C (stationary phase) and killed by heat treatment at 65 °C for 20 min. Killed bacteria were washed thrice in PBS before being resuspended to approximately 1 × 10^8^ cfu/mL in IMDM media again supplemented with L-glutamine, 20% FBS and 1 X Antibiotic-Antimycotic. Ex vivo bovine milk CD14^+^ cells were then challenged with 1 × 10^7^ cfu/mL (110 µL of bacteria suspension) of killed *S. uberis*. This equates to a 10:1 multiplicity of infection of bacteria:CD14 macrophage. Then, 210 µL of media was removed for the T0 timepoint. At 24 h, the remaining media was removed for the T24 timepoint and CD14^+^ cells were frozen in Trizol for subsequent RNA extractions.

Challenges with WT *S. uberis* were also performed in the presence of cell-permeable inhibitors Z-VAD-FMK (Sigma, UK) (100 μM), Ac-YVAD-cmk (Sigma, UK) (50 μM), MCC950 (Stratech Scientific, UK) (1 µM), or Cytochalasin D (Stratech Scientific, UK) (10 µM) dissolved in DMSO. These were performed alongside challenge with WT *S. uberis* and DMSO, no bacterial treatment with DMSO, or SUB1154-deletion and DMSO.

Recombinant SUB1154 protein was expressed and purified from *E. coli* strain M15 pREP4 expressing the SUB1154 protein in a pQE1 vector as reported previously [31]. Briefly, following IPTG induction, the protein was expressed overnight at 18 °C before extraction. Following HIS column purification, the protein was dialysed for a total of 4 h at 4 °C to remove imidazole. Pierce High Capacity Endotoxin removal columns (Thermo Scientific, UK) were used before a final concentration and clean-up step using Amicon Ultra columns (Merck, city, country) (100 KDa) was performed. As with the previous cell challenges, a 12-well dish was seeded with 1 million CD14^+^ cells per well and allowed to recover overnight. Adherent cells were washed with pre-warmed (37 °C) PBS and given fresh growth media 1 h before challenge with WT *S. uberis*, 0.02 μM recombinant 1154 protein alone, SUB1154 deletion *S. uberis*, or a mix of SUB1154 deletion *S. uberis* and 0.02 μM recombinant 1154 protein. We also challenged macrophages with the deletion strain and incubated the cells for 12 h before adding the recombinant SUB1154 protein to a final concentration of 0.02 μM.

ELISA for bovine IL-1β was then performed using the same uncoated ELISA kit as for the milk ELISAs. Additionally to ANOVA (Table S4), student’s *t*-test was performed for direct comparisons indicated in the figures. 

### 4.11. Extraction, Purification, and Library Preparation of mRNA from Challenged CD14^+^ Leukocytes

Challenged ex vivo cells at either T0 or T24 were washed vigorously with 200 µL ice-cold PBS to release them from the tissue culture surface and removed immediately to a 1.5 mL microfuge tube containing 800 µL Trizol (Invitrogen, Carlsbad, CA, USA). Three biological replicates of CD14^+^ leukocytes treated with WT or SUB1154 deletion strains of *S. uberis* 0140J were generated at both T0 and T24. The cell-Trizol mixtures were vortexed and frozen at −80 °C. After thawing, RNA was chloroform-extracted from Trizol and precipitated overnight at −20 °C with 1 volume of isopropanol, 0.1 volume of 3 M sodium acetate, and 1 µL of glycogen. After centrifugation for 30 min at 20,000× *g*, the pellet was washed with 70% ethanol and vacuum-dried. The total RNA pellet was then resuspended in 10 µL of nuclease-free water, and concentration was determined with a Nanodrop (Thermo Scientific, city, country). Libraries were generated for Illumina sequencing using the Quant-seq 3′-FWD strategy (Lexogen, Vienna, Austria). The manufacturer’s instructions were followed with the addition of the UMI second-strand synthesis module for QuantSeq FWD, which adds unique molecular identifiers to each molecule during second-strand synthesis (step 7 of the protocol), to enable bioinformatic correction of bias introduced by library preparation methods [51]. Eighteen PCR cycles were used during the final multiplexing PCR.

### 4.12. Illumina Sequencing and Differential Gene Expression Analysis

The libraries were pooled after Qubit cDNA quantification with an equimolar concentration (4 nM) targeted for the 12 libraries to be included in differential gene expression analysis.

The final pool was quantified by Qubit, Tapestation, and KAPPA library quantification kit (Illumina) before final preparation and loading as prescribed by NextSeq System Denature and Dilute libraries guide (Illumina).

Following sequencing on the Illumina nextseq 500, basecalling was performed with BCL2FASTQ. Adding the UMI to read names, trimming, alignment, and collapsing the UMIs to the BAM files was performed using the bioinformatics pipeline as provided by Lexogen and BlueBee genomics. In brief, the first 6 nucleotides of read 1 constitute the unique molecular identifier which are added to the read name before trimming with bbduk. Trimmed reads were aligned to the bovine UMD_3.1.1 genome with STAR [73]. UMI-collapsed BAM files were indexed with samtools_index before gene counting was performed with Htseq-count [74]. Read counts were read into R studio (ver. 1.2.5019, R Studio, Inc., Boston, MA, USA) for differential expression analysis with DESEQ2 [75].

Two types of differential expression analysis were performed as described in the DESEQ2 vignette. Simple “A vs. B” analysis was performed to compare T0 and T24 gene expression in CD14^+^ leukocytes challenged with WT *S. uberis* and SUB1154-deletion *S. uberis* and to directly compare T24 timepoints between the deletion and WT. A multifactor differential expression analysis was performed with genotype and timepoint as factors to compare genes differentially expressed at T24 in WT with those genes differentially expressed at T24 in response to the deletion strain, thereby isolating the changes caused in response to strains expressing WT SUB1154 protein. GO term and reactome enrichment was performed using panthergo [76].

### 4.13. Data Visualisation

Redundant GO terms were manually removed from Figures but not supplementary data. Data visualisation was carried out with GraphPad Prism (ver. 8.3.0, GraphPad Software, LLC, San Diego, CA, USA) and the R packages EnhancedVolcano Version 1.4.0 [77] and ggplot2 [78] in the R studio environment. Network analysis and Figures were drawn using NetworkAnalyst 3.0 [79]. KEGG pathway maps [80] were downloaded and annotated using pathview [81] in R studio. Schemas (i.e., Figure 2A and Graphical Abstract) were created with BioRender.com.

### 4.14. Quantification and Statistical Analysis

Data were combined for charts and presented as mean ± SEM. Data were normally distributed; thus, unless otherwise stated, differences between genotypes along timepoints were assessed by repeated-measures two-way analysis of variance (ANOVA), with genotype and time as factors and using the Geisser-greenhouse correction for sphericity and the genotype factor *p*-values reported in the figure legends. Per-timepoint variances were computed for each genotype and compared with the mean for response to WT for the same timepoint, and uncorrected Fisher’s least significant difference (LSD) reported in Table S4 using GraphPad Prism ver. 8.3.0. Differential expression analysis data were tested for significance using the default DESEQ2 settings, thus using a Wald test for *p*-value and Benjamini–Hochberg procedure for generation of adjusted *p*-values.

### 4.15. Data and Code Availability

The RNA-seq differential expression analysis has been deposited on the gene expression omnibus (GSE138793). Uninterpreted outputs of differential expression, gene ontology, and reactome enrichment are in Supplemental Data (Tables S1–S3). Values used for calculations of means in Figure 1; Figure 2 are in the Supplemental Data (Table S4).

### 4.16. Materials Availability

Reagents or further information may be obtained from the lead contact James Leigh (james.leigh@nottingham.ac.uk).

## Figures and Tables

**Table 1:** Mastitis clinical scoring used for animal challenge studies.

*Score*	*Appearance of Quarter*	*Score*	*Appearance of Milk*
1	Normal	1	Normal
2	Minor changes (e.g., hardness)	2	Minor changes (e.g., a few flakes)
3	Moderate signs (e.g., heat, tenderness)	3	Moderate signs (e.g., clots, clumps)
4	Severe signs (e.g., distended and discomfort on palpation)	4	Severe Signs (e.g., changes in colour, composition)

## Supplementary Materials

The following are available online at https://www.mdpi.com/2076-0817/9/12/997/s1. Figure S1: Gross transcriptome comparisons; Figure S2: log_2_ Fold-Change of genes associated with Inflammatory response; Figure S3: Network analysis on genes upregulated in response specifically to wild-type sub1154 protein; Figure S4: KEGG pathway maps; Figure S5: Flow cytometry of cells purified from milk and further enriched with flowcomp CD14+ beads; Figure S6: Top 10 up- or downregulated genes in CD14+ cells; Table S1: Differential gene expression results of macrophage responding to *S. uberis* wildtype; Table S2: Differential gene expression results of macrophage responding to *S. uberis* SUB1154-Deletion; Table S3: Differential gene expression results of macrophage responding to the presence of SUB1154 protein; Table S4: Bacteriology and ELISA data.
