# Supplementary material for: A Paradox in Bacterial Pathogenesis: Activation of the Local Macrophage Inflammasome Is Required for Virulence of Streptococcus uberis
Source: Pathogens. 2020 Nov 28;9(12):997. doi: 10.3390/pathogens9120997 (PMC7768481; doi:10.3390/pathogens9120997)
Supplement: Supplementary file 1 [file pathogens-09-00997-s001.zip › Captions for supplemental files.docx]

**Captions for supplemental files**

**Figure S1 Gross transcriptome comparisons**. Log Fold Change and Mean of Normalised Counts were plotted for each treatment to generate MA plots where grouping along the red line denotes similarity as seen in wildtype compared to deletion, both at T0 (C). Comparisons were generated for all A_B treatment groups tested. A heatmap of the top 20 genes upregulated or downregulated at the 24 hour timepoint compared to T0 (E) is shown for each genotype plus the multifactor differential expression analysis.

**Figure S2 Log2 Fold Change of genes associated with Inflammatory response**, all were significant, positive DEGs in wildtype (WT) (l2fc >1.5, padj <0.05) and these genes used to call l2fc values from deletion (Del) differential expression results regardless of adjusted pvalue. Lowest L2FC on scale is -1.

**Figure S3 Network analysis on genes upregulated in response specifically to Wildtype sub1154 protein** (>1.5 l2fc, p<0.05). Proteasome complex in yellow. (GO CC query on all nodes padj 8.31e-49). Phagocytosis in blue (padj 1.54e-10). In black is regulation of cytokine biosynthetic production (padj 0.000867) and in red, positive regulation of cytokine secretion (padj 0.000867). Modules with large populations of transcription and DNA Topology, translation, and Interleukin-1 receptor associated Kinase (IRAK-1) were manually annotated.

**Figure S4. KEGG pathway maps of cytokine-cytokine receptor interaction (A) and Cell Adhesion Molecules (B)** showing DEGs with p ≤0.05 coloured according to log2fold changes in response to the presence of sub1154 protein according to the multifactor differential expression analysis.

**Figure S5. Flow cytometry of cells purified from milk and further enriched with flowcomp CD14+ beads**. Neutrophils are detected as populations of positive Ly6G-gr1, positive CD45, and high side scatter (more granular cells). Non-neutrophils are detected by high CD45 and lower side scatter (i.e. monocytes).

**Figure S6. Top 10 up (A,C) or downregulated (B,D) genes in CD14+ cells challenged with Wildtype (A,B) or SUB1154-Deletion (C,D) strains of S. uberis.**

**Table S1. Differential gene expression results of macrophage responding to S. uberis wildtype.** These are the raw data used for Figure 3A and Figure 3C. Shown also are raw GO term and Reactome enrichment analysis results used for Figure 4.

**Table S2. Differential gene expression results of macrophage responding to S. uberis SUB1154-Deletion.** These are the raw data used for Figure 3B and Figure 3C. Shown also are raw GO term and Reactome enrichment analysis results used for Figure 4.

**Table S3. Differential gene expression results of macrophage responding to the presence of SUB1154 protein.** Shown also are raw GO term and Reactome enrichment analysis results. These data were generated using the multifactor differential gene expression analysis, represented in Figure 5.

**Table S4 Bacteriology and ELISA data.** Tables of values from bacteriology and somatic cell count in vivo (A), IL-8 (B), IL-1β (C) and IL-6 (D) in response to quarter challenge with S. uberis Wildtype, SUB1154-Truncated, and SUB1154-Deletion. Used in the construction of Figure 1. Also here are ex-vivo results of macrophage enriched populations responding to S. uberis Wildtype, SUB1154-Truncated, and SUB1154-Deletion. Showing IL-1β responses to strain (E), in the presence of Caspase inhibitors (F), MCC950 and Cytochalasin D (G), or recombinant protein (H), as used in the construction of Figure 2. Cathelicidin (I) and BSA (J) measurements are presented here, as used in the construction of Figure 1.
