# Supplementary figures and images for: A Paradox in Bacterial Pathogenesis: Activation of the Local Macrophage Inflammasome Is Required for Virulence of Streptococcus uberis
Source: Pathogens. 2020 Nov 28;9(12):997. doi: 10.3390/pathogens9120997 (PMC7768481; doi:10.3390/pathogens9120997)

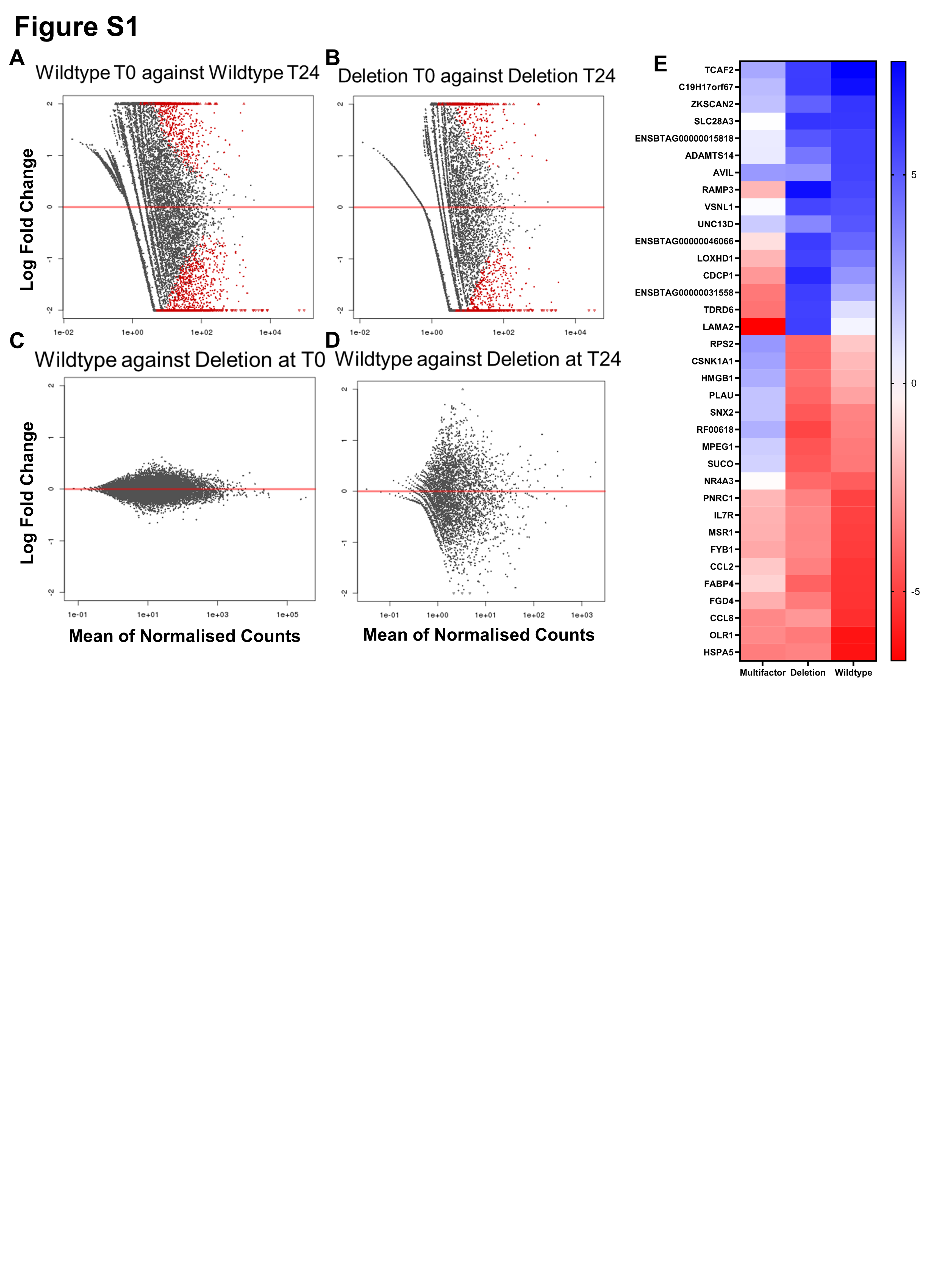

Supplement: Supplementary file 1 [file pathogens-09-00997-s001.zip › Figure S1.tif]

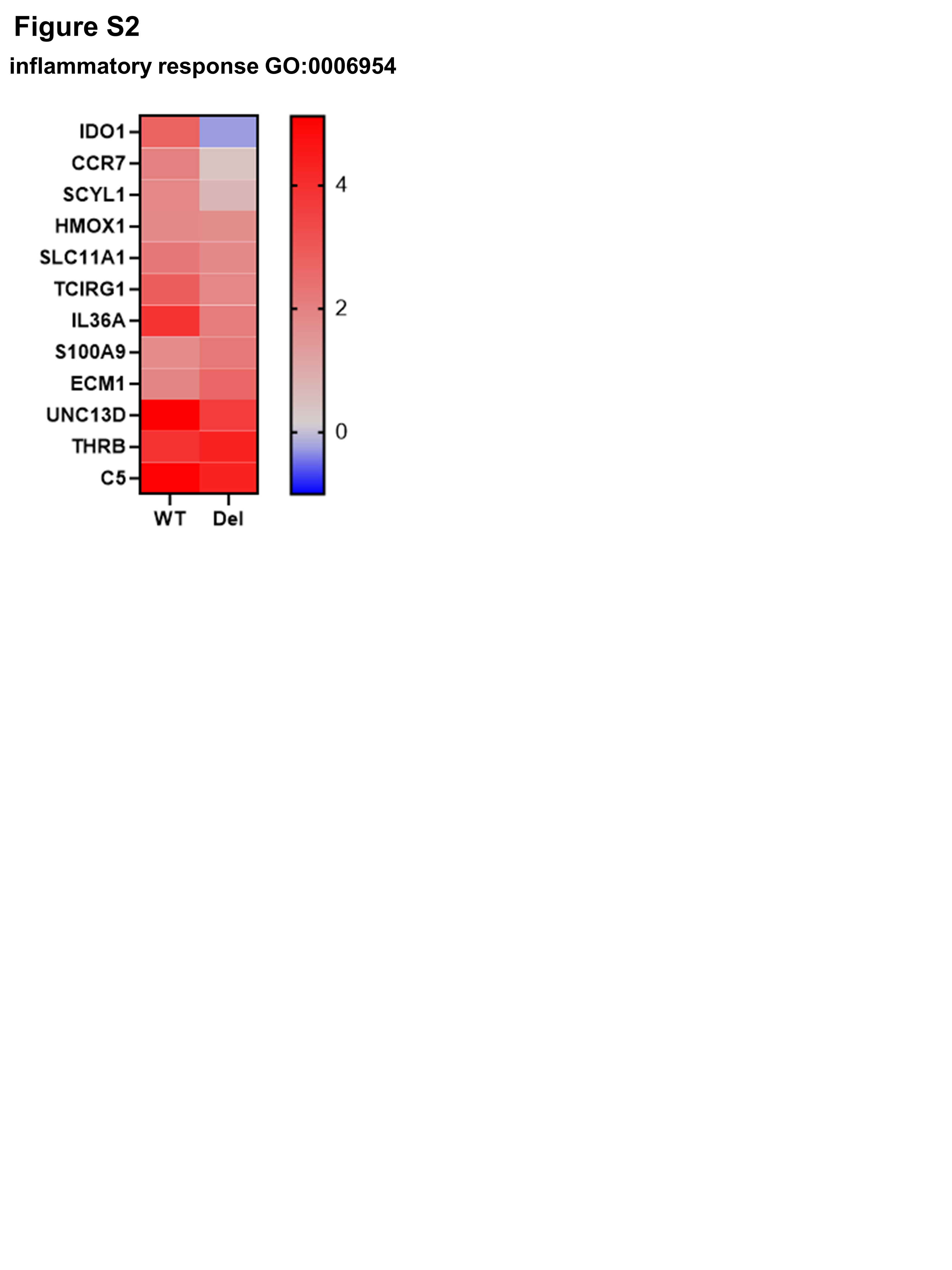

Supplement: Supplementary file 1 [file pathogens-09-00997-s001.zip › Figure S2.tif]

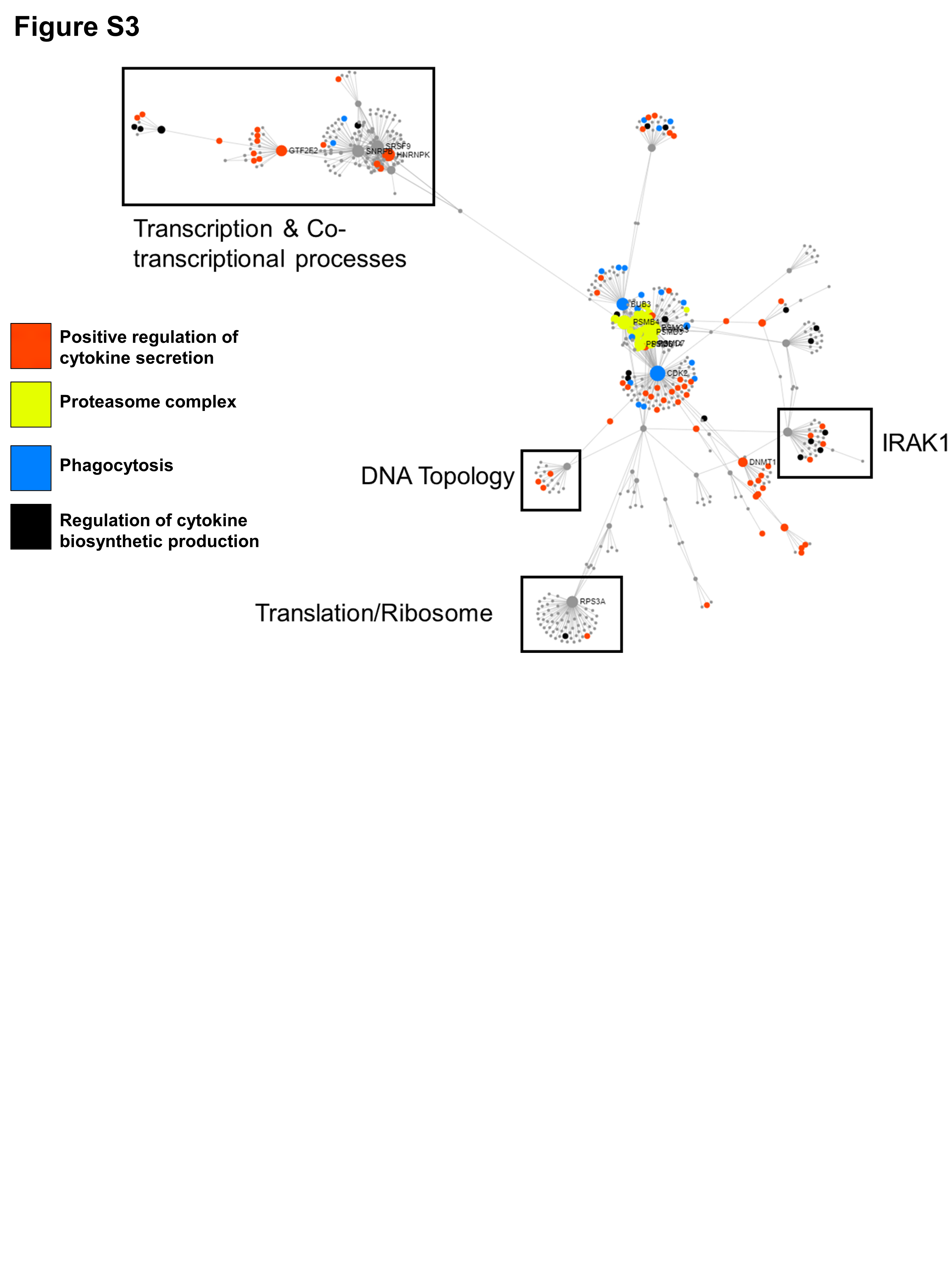

Supplement: Supplementary file 1 [file pathogens-09-00997-s001.zip › Figure S3.tif]

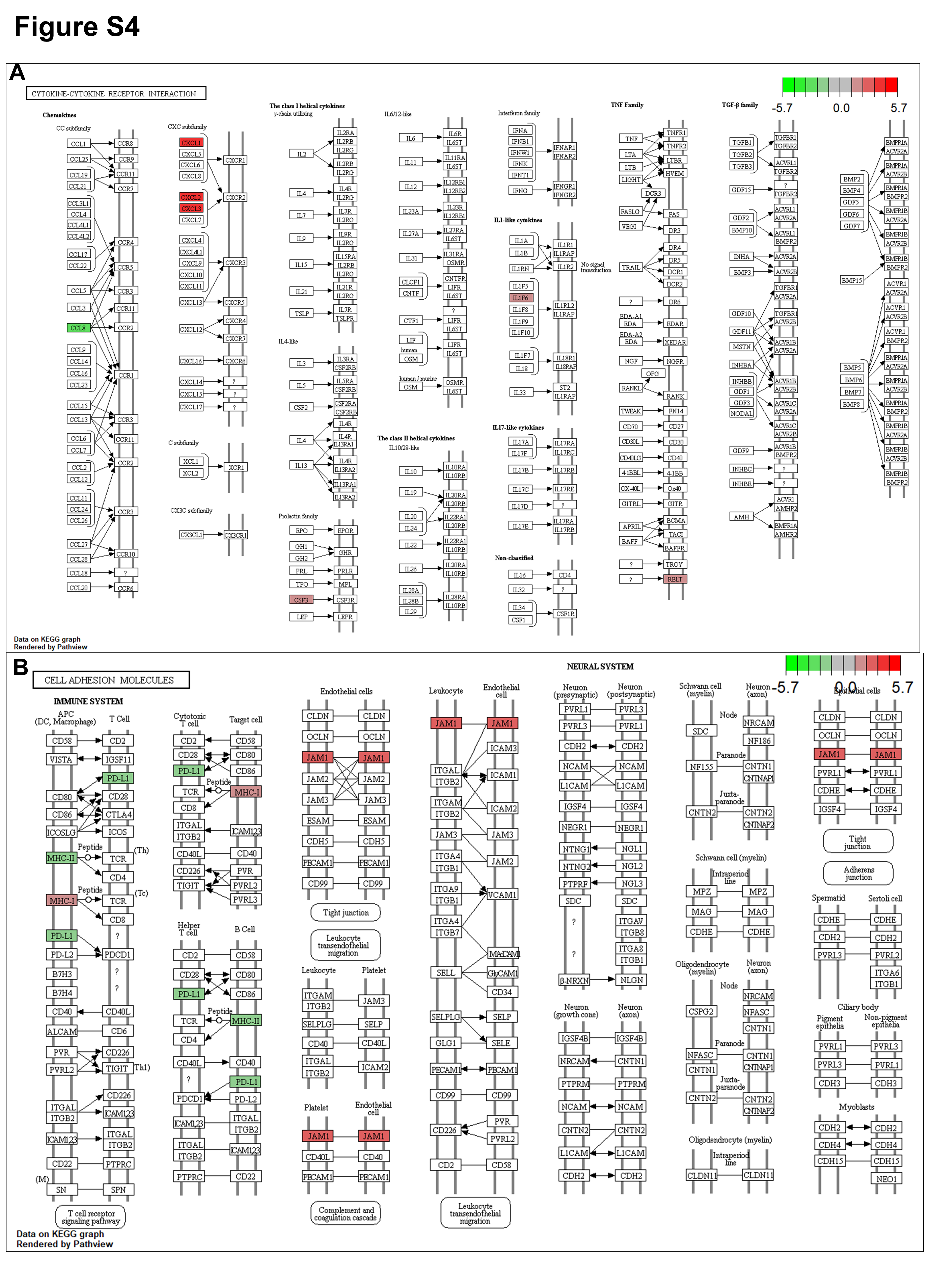

Supplement: Supplementary file 1 [file pathogens-09-00997-s001.zip › Figure S4.tif]

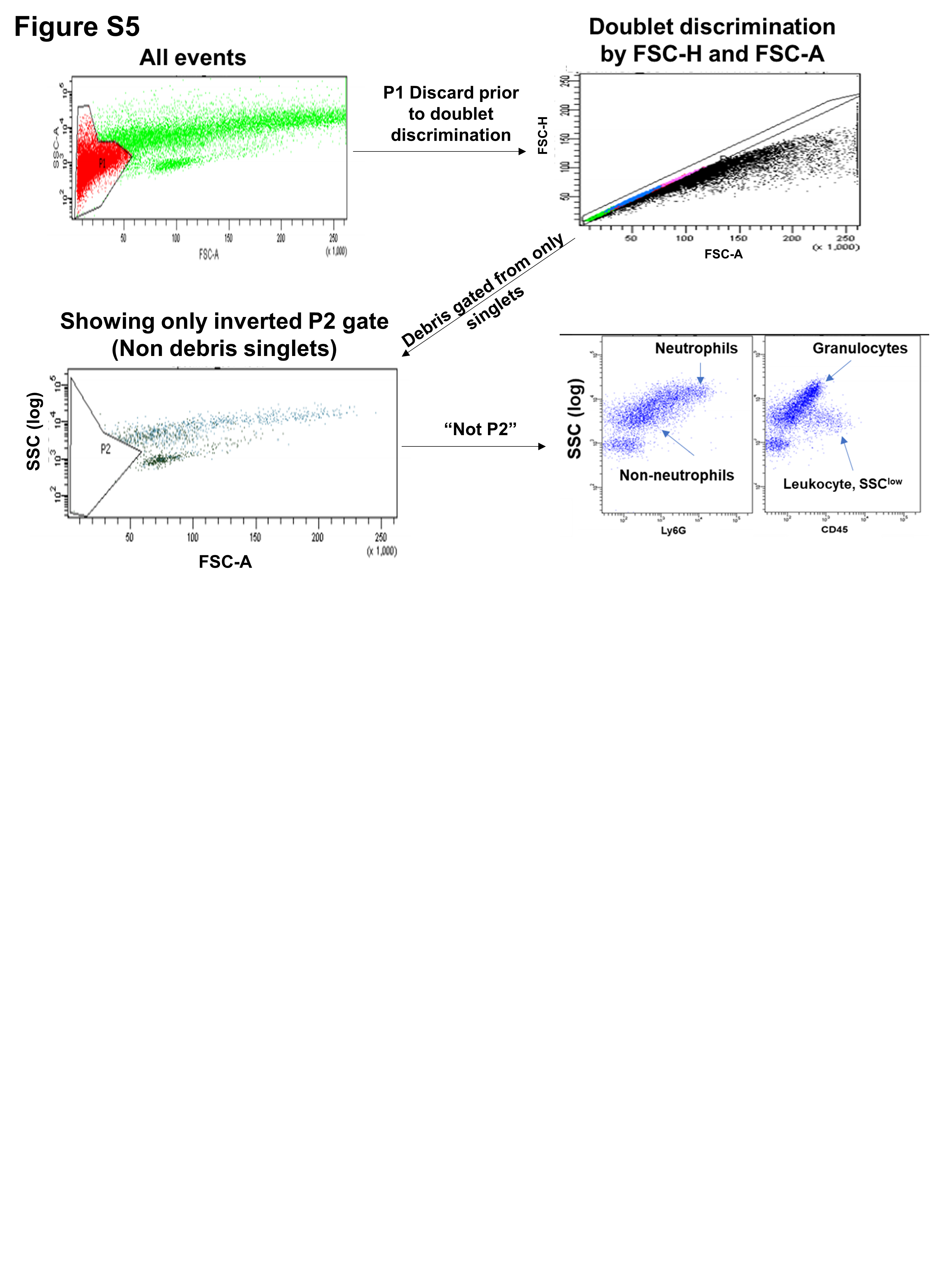

Supplement: Supplementary file 1 [file pathogens-09-00997-s001.zip › Figure S5.tif]

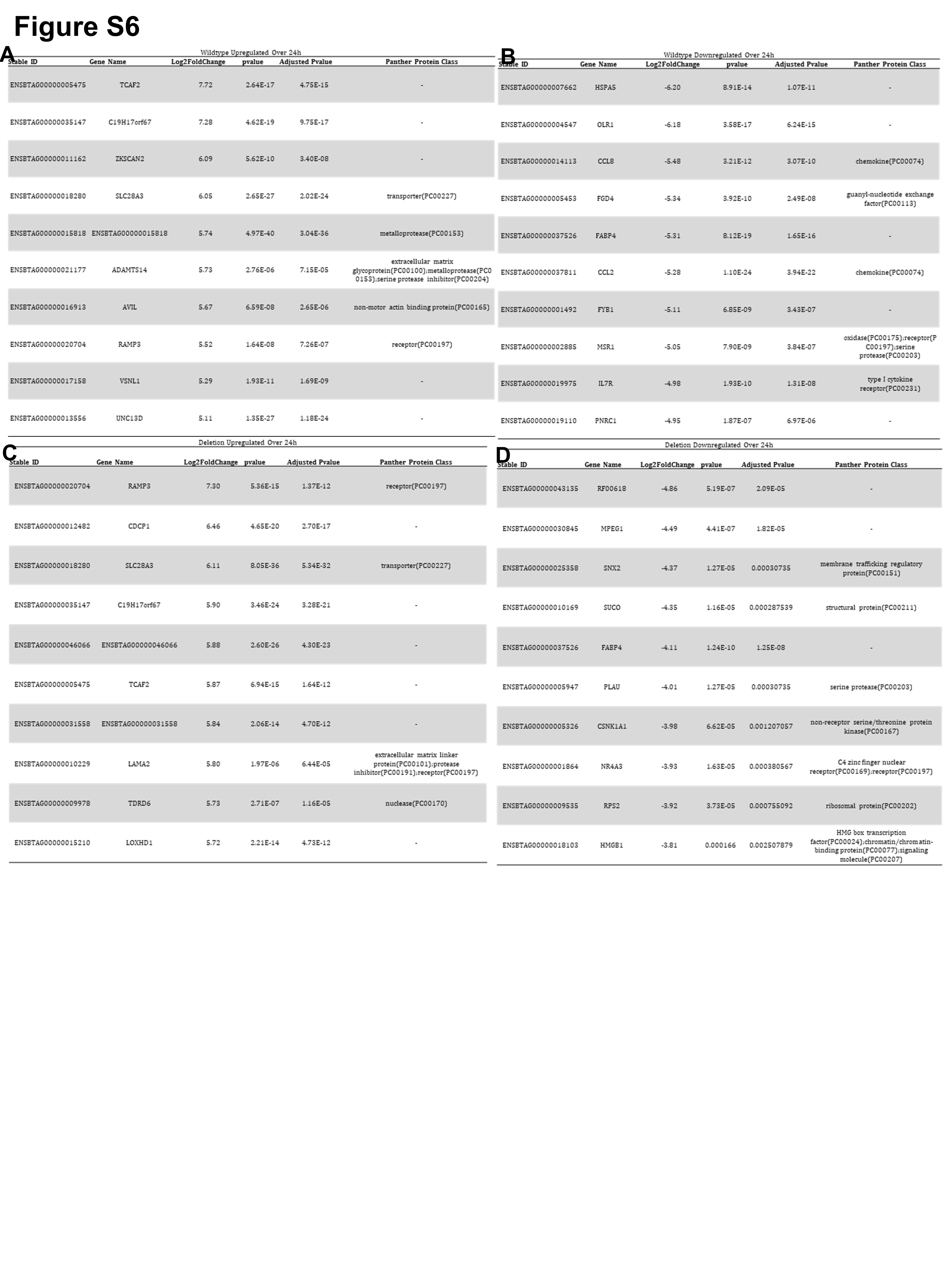

Supplement: Supplementary file 1 [file pathogens-09-00997-s001.zip › Figure S6.tif]
